# What is the role of individual accountability in patient safety? A multi‐site ethnographic study

**DOI:** 10.1111/1467-9566.12370

**Published:** 2015-11-04

**Authors:** Emma‐Louise Aveling, Michael Parker, Mary Dixon‐Woods

**Affiliations:** ^1^ Department of health SciencesUniversity of LeicesterUK; ^2^ Department of Health Policy and Management, Harvard T.H. Chan School of Public HealthBostonUSA; ^3^ Ethox CentreUniversity of OxfordUK; ^4^ Department of health SciencesUniversity of LeicesterUK

**Keywords:** safety, ethnography, patient and public engagement

## Abstract

An enduring debate concerns how responsibility for patient safety should be distributed between organisational systems and individual professionals. Though rule‐based, calculus‐like approaches intended to support a ‘just culture’ have become popular, they perpetuate an asocial and atomised account. In this article, we use insights from practice theory – which sees organisational phenomena as accomplished in everyday actions, with individual agency and structural conditions as a mutually constitutive, dynamic duality – along with contributions from the political science and ethics literature as a starting point for analysis. Presenting ethnographic data from five hospitals, three in one high‐income country and two in low‐income countries, we offer an empirically informed, normative rethinking of the role of personal accountability, identifying the collective nature of the healthcare enterprise and the extent to which patient safety depends on contributions from many hands. We show that moral responsibility for actions and behaviours is an irreducible element of professional practice, but that individuals are not somehow ‘outside’ and separate from ‘systems’: they create, modify and are subject to the social forces that are an inescapable feature of any organisational system; each element acts on the other. Our work illustrates starkly the structuring effects of the broader institutional and socioeconomic context on opportunities to ‘be good’. These findings imply that one of the key responsibilities of organisations and wider institutions in relation to patient safety is the fostering of the conditions of moral community.

## Introduction

Though more than 15 years have passed since the birth of the modern patient safety movement (Kohn *et al*. 2000), one of its most important debates endures: how to distribute responsibility between organisational systems and individual professionals. The early phase of the movement was dominated by the view that error was not the result of individual failing, but instead was an inescapable feature of poorly designed systems. Accordingly, it was argued individuals should not be blamed for safety lapses: the proper response was said to involve the re‐engineering of systems to avert or mitigate error (Leape *et al*. 2000). More recently, this so‐called ‘systems’ approach has been argued to result in an unwarranted, misguided and risky attribution of all responsibility for safety to systems (Wachter and Pronovost 2009). A ‘just culture’ rather than a no‐blame approach is now increasingly advocated, amid calls for individuals and systems both to be accountable and for those accountabilities to be balanced (Wachter 2013).

The question of how to allocate responsibility between systems and individuals has important instrumental value: it is of critical practical relevance because getting it wrong may undermine safety. Disciplining individuals who make errors in contexts of inadequately designed or poorly functioning systems may occlude deep organisational or institutional pathologies. Searching for systems defects when an individual is at fault may be an equally fruitless effort. Yet current prescriptions for the making of judgements to support a just culture draw upon only a limited evidence‐base (empirical and theoretical) and tend to be prescriptive and mechanistic. One widely‐cited ‘algorithm’ for determining the responsibility of individuals, for example, distinguishes between three types of error (human error, reckless behaviour, and at‐risk behaviour) and matches them to a proposed response (consoling, punishment, and coaching, respectively) (Marx 2001). Another decision‐tool uses a ‘culpability tree’ (Meadows *et al*. 2005) to guide users through a series of questions about the individual's actions, motives and behaviour at the time of the incident. These formulas have been criticised for their essentialist assumption that some acts or behaviours are inherently culpable and for their supposition that the making of distinctions between the acceptable and the unacceptable can be rendered tractable to simple rules (Dekker 2012). They may be therefore ill‐suited even to the instrumental task of improving the effectiveness of patient safety efforts.

The problems with this calculus‐like approach go far deeper, however. First, in their preoccupation with instrumental value, they tend to diminish the intrinsic value of an explicit emphasis on the moral agency of individuals. The idea that there is an inherent good in asking people to be good goes back to antiquity, but it is one that has special valence for the healthcare professions. The term ‘profession’ has been linked to virtues – such as benevolence, compassion, mercy and competence – since the earliest usage of the term (Pellegrino 2002). Recent years have seen renewed sociological attention – from previously sceptical quarters – to the social function and value of a morally‐founded conceptualisation of the professions, accompanied by warnings of the dangers of its diminishment (Brint 2006, Freidson 2001).

Second, calculus‐like approaches promote an asocial, atomistic and static account, one that neglects long‐standing sociological insights about the scope, nature and possibilities of the individual agency of situated actors in institutionalised settings. In the field of organisation studies, such insights are increasingly gathered under the rubric of ‘practice theory’, which promotes an understanding of organisational phenomena as ‘dynamic and accomplished in ongoing, everyday actions … we understand the mutually constitutive ways in which agency is shaped by but also produces, reinforces and changes its structural conditions’ (Feldman and Orlikowski 2011: 1250). In offering this account of the emergent constitution of the social world through routine practices in organisations, practice theory explicitly invokes a rich sociological heritage, including (though not only) Giddens's (1984:26) conceptualisation of structure and agency as a duality, mutually reinforcing and in constant dynamic interaction, such that the ‘moment of production of action is also one of reproduction’. On this view, structure creates and shapes the possibilities for agency, at the same time as agency creates and shapes structure.

Positioning individuals as knowledgeable agents who reflexively monitor the flow of interactions with one another, Giddens (1984: 30) introduces a notion of accountability that emphasises the answerability of actors in terms of norms: ‘To be “accountable” for one's activities is both to explicate the reasons for them and to supply the normative grounds whereby they may be “justified”’. He also notes that such norms cannot readily be programmed externally (for example through codes of conduct); instead normative expectations are socially contingent and must be sustained through the effective mobilisation of sanctions during actual encounters. Accordingly, for actors in specific social environments, what is deemed right and proper conduct is likely to be far more influenced by norms and values as they are produced and reproduced within those environments than they are by external standards and codifications. For those seeking to examine patient safety, a critical set of tasks therefore focuses on characterising how the work of healthcare gets done, how the norms, routines and institutionalised practices of organisational settings allocate responsibility and facilitate distinctions between blameless and blameworthy actions, and how, by whom and to whom the available sanctions are applied.

These are the tasks that *Forgive and Remember*, Bosk's (2003) classic ethnography assumes. Though he does not use the term ‘practice theory’ explicitly (the term was developed subsequent to his work), Bosk's study of surgeons‐in‐training vividly demonstrates the salience of that literature. He identifies how norms of responsibility are articulated and enforced through repeated and collectivised patterns of noticing, recognising, explaining, and disciplining actions and events. He shows how individuals are made accountable for what they do through processes of social control that, crucially, do not shrink from the imputation of blame: some errors may be deemed ‘forgivable’ but others taken as evidence of moral failing. Among the less forgivable errors are those that fail to honour the commitments that the profession requires; these errors are both sanctionable and sanctioned.

In calling out the importance of blame and punishment, *Forgive and Remember* disrupts the narrative of default blamelessness associated with the systems approach to patient safety, but it continues a sociological tradition dating back to Durkheim about the value of sanctioning as a collective responsibility that helps to make visible and reinforce the norms of a community (including a professional community) and to increase solidarity with that community. Bosk also makes another crucial, and under‐recognised, observation. He shows that while near‐universal consensus may exist on the culpability of some behaviours and actions, another class of apparent violations – termed ‘quasi‐normative’ errors – involves failure to comply with senior physicians’ personal preferences. This apparently more capricious category makes the broader point that situated agents may not themselves agree on what constitutes good practice. If calculus‐like approaches are limited by their simplistic and flawed assumptions, and leaving it up to agents in their own environments susceptible to arbitrariness, then alternative ways of reasoning about how to draw boundaries around the accountabilities of individuals are needed. We suggest that concepts and reasoning from the ethics and political science literatures have much to offer in this regard.

A first and basic question concerns the extent to which individuals qualify as having responsibility for which they are answerable (and are thus accountable). We propose that to be held accountable, a moral agent must know of the standards she is expected to meet, be charged with responsibility for meeting those standards, and have sufficient autonomy and capacity in her choice of actions, and access to resources, to be able to comply: ‘ought implies can’ (Kant 1973). Assessments of accountability thus need to be attentive to the constraints on choices and actions, and to the nature of those constraints.

A second question concerns how to identify individual contributions to patient safety given that the potential contributors may be multiple and widely diffused, for example across teams, organisations (and their internal strata and divisions), and wider institutions (Bell *et al*. 2011). Patient safety is thus an example of the more general phenomenon known as the ‘problem of many hands’ (Thompson 1980). Described by the political philosopher Dennis Thompson, it applies to situations where many people contribute in many different ways to particular outcomes, so that the ‘profusion of agents obscures the location of agency’ (Thompson 2014: 1). Thompson offers two criteria that clarify individual moral responsibility in a collectivity:

1the individual's actions or omissions make a causal contribution to the outcome and
2these actions or omissions are not done in ignorance or under compulsion.

These criteria might best be understood as necessary but not sufficient conditions, such that individuals should be candidates for being held accountable for any actions or omissions only if they are met. In a healthcare context, a promising approach to augmenting these basic qualifying criteria is offered by the physician and ethicist Edmund Pellegrino (2004). Rejecting a no‐blame system as a travesty of social and commutative justice, and emphasising the interdigitation and ethical reciprocity of individual and collective virtue, he proposes four major organising principles:

1a properly organised organisational and systemic context is essential to reduce the prevalence of healthcare error;
2its effectiveness and efficient working depend on a parallel affirmation of the moral duty and accountability of each health professional in the system;
3each individual health professional must possess the competence and character crucial to the performance of her particular function as well as those of the system as a whole; and
4the major function of a system is to reinforce and sustain these individual competencies and virtues.

For an accountability system to function, criteria and principles alone are not enough, however: also required is a structural arrangement that can make clear the relevant expectations and standards, define the actors that have responsibility for meeting those expectations and standards, create a forum to whom those actors are answerable, and enable the forum to pose questions, pass judgement, and impose consequences on the actors (Bovens 2007).

We propose that, taken together, Thompson's criteria and Pellegrino's principles, along with an understanding of the structural requirements of an accountability system, provide a potentially useful framework for structuring thinking about questions of individual responsibility and its intersection with systems. Yet, as practice theory makes clear, such a framework is, by itself, likely to be sterile in the absence of empirical evidence. In this article, we use the framework as a starting point for analysis of the role of personal accountability for patient safety using ethnographic data from contrasting hospital contexts.

## Methods

We conducted ethnographic case studies of five large acute hospitals (Table 1): two (Sukutra and Nikalele) in two low‐income African countries and three (Farnchester, Greenborough and Worpford) in England, a high‐income setting. These case studies were selected from two research projects with similar aims and design. Four cases – two in England and two in Africa – were drawn from Project 1, which examined quality and safety in high and low‐income countries. The data collected from the English sites was less extensive than from the African sites, so one case was augmented using data from its participation in Project 2, a study of culture and behaviour related to quality and safety in the English NHS (Dixon‐Woods *et al*. 2013). A further English case was also selected from Project 2, yielding two African case studies and three UK case studies in total.

**Table 1:** *Details of the five ethnographic case studies*

Site	Interviews	Observations	Staff roles and areas of observation
Sukutra hospital Location: Africa Teaching, referral hospital	30 individuals	30 days (131 hours)	Physicians, nurses, midwives, clinical technicians, senior and middle managers, administrative staff. Surgical, neonatal, maternity services
Nikalele hospital Location: Africa Teaching, referral hospital	31 individuals	46 days (177 hours)	Physicians, nurses, midwives, clinical technicians, senior and middle managers, administrative staff. Surgical, neonatal, maternity services
Farnchester hospital Location: UK Teaching, referral hospital	32 individuals	41 days (252 hours)	Physicians, nurses, operating room and administrative staff, senior and middle managers. Surgical, neonatal, renal, infection prevention and control services and emergency departments.
Greenborough hospital Location: UK Teaching, referral hospital	13 individuals	6 days (38 hours)	Physicians, nurses, clinical technicians, senior and middle managers, administrative staff. Surgical, neonatal, infection prevention and control services.
Worpford hospital Location: UK Teaching, referral hospital	20 individuals	9 days (66 hours)	Physicians, nurses, ODPs, healthcare assistants, midwives, senior and middle managers. Surgical and maternity services.
Total:	126 individuals	132 days (664 hours)	

Ethical approval was obtained from each of the African sites and separately for the English sites. Further details are not provided in order to protect the anonymity of the sites. For the same reason, hospitals are given pseudonyms, and quotation labels give minimal identifying information (site and professional role). At the request of the research participants, the countries in which African sites were located have not been named. Thus, while we do not intend to imply an unwarranted degree of similarity across different African countries, we are restricted in the healthcare system details we can provide. What can be reported is that both African hospitals were government‐run, teaching referral hospitals located in towns, serving a mixed urban‐rural population. All three UK hospitals were large NHS teaching hospitals located in cities and serving as tertiary centres for a wider region.

With the verbal permission of staff and, where appropriate, that of patients, more than 660 hours of wide‐ranging non‐participation observation were undertaken in diverse areas of the five hospitals (76 days in Sukutra and Nikalele; 56 days in the English hospitals) covering managerial and clinical meetings as well as clinical activity. Interviews (some group‐based) were conducted with informed consent from 124 hospital staff (Table 1) and were digitally recorded, translated where necessary and transcribed verbatim. At the interviewees’ request, two other interviews were not recorded; notes were taken instead. Recruitment of participants was guided by purposive sampling to ensure diversity in terms of seniority, role, profession, subspecialty and area of practice. Interview topics covered perceptions of influences on and challenges of achieving patient safety.

Analysis of data was based on the constant comparative method (Charmaz 2006), which was informed, but not constrained, by sensitising constructs derived from the research questions and the relevant literature. The initial framework (based on Pellegrino and Thompson) proved very useful for structuring thinking about accountabilities of individuals and systems, but also required modification in light of the empirical findings. Supported by NVIVO software (QSR International, Brisbane), ELA led the analysis of data, including development of a coding scheme applied across all transcripts. MDW and MP reviewed samples of coded extracts at different stages and helped to refine analytic categories. By comparing and contrasting cases from diverse contexts, we sought to move beyond description of differences to theoretical insights (Druckman 2005). Conducting this kind of analysis involves multiple sensitivities, especially when it involves such diverse contexts of resources, history and environments. In order to avoid pathologising any particular setting or group, it is important to stress that our findings should not be read as the essentialist traits of any particular society, country or professional grouping.

## Findings

Our observations and interviews across the five sites repeatedly confirmed the collective nature of the healthcare enterprise and the extent to which patient safety depended on contributions from many hands. For patients to remain safe, multiple interacting microsystems – from equipment design, maintenance and supply through administrative procedures to the performance of clinical practices, and much else – needed to go right. The extreme interdependence of each individual upon others meant that the individual who was most proximal to a specific poor outcome or ‘near miss’ was only rarely solely responsible. (For example, on one occasion it was observed that a nurse in Sukutra had forgotten to administer prophylactic antibiotics before surgery; however, as we go on to show, such errors were arguably a reflection of more systemic challenges within the organisational context.) But rarely too was their contribution completely negligible, and sometimes a particular individual's efforts were essential to preventing harm (for example, a neonatal nurse in an English site raising the alarm about monitoring equipment that did not appear to be functioning properly). Further, we found that an approach that focused solely on specific incidents or events offered only a partial and misleading account; consistent with practice theory, individuals also contributed to the prevailing conditions and environments for safety through the norms they produced and reproduced and through their behaviours and demonstration of professional virtues. Thompson's criterion that individuals should make a causal contribution to safety was thus easily met much of the time, though the extent to which any individual was the single or most important cause was highly variable.

Thompson (1980: 909) emphasises that individuals cannot be accountable for actions and omissions done in ‘ignorance’, including ‘the formal and informal expectations of the individual's official role’. But across the five hospitals, we found that the standards that individuals should meet – whether of practice or conduct – were not always clear to them, that official standards were heavily distorted by customary practice, and that multiple and sometimes competing conceptions of safe practice were in play. As Giddens anticipated, formal guidance played an ambivalent and unstable role as a source of standards for practice. One basic but pervasive problem concerned workers’ awareness of the relevant rules. In the African hospitals, clinical and administrative protocols were often lacking entirely (though more were gradually being introduced) or workers lacked knowledge of those that did exist because they had not been trained. Most clinical areas lacked protocols for appropriate antibiotic prescribing, for example, although in one site work was being undertaken with outside experts to develop local protocols for the neonatal and surgery departments. In the English hospitals, the problem was not so much too few protocols as too many, and policies that changed too frequently: workers reported that it was simply impossible to keep up to date, partly because of cuts to training:

That induction part is not there, you will just be employed and you will be assigned to one ward and you start working with the people there. [Manager, Nikele]

Six months down the line the staff that needed educating haven't been taught what they needed to know. [Manager, Worpford]

The challenges of making clear to people what was expected of them went well beyond formal standards, however. What individuals saw themselves as responsible for was profoundly shaped both by organisational contexts – which were typically rich in operational and managerial defects – and by the prevailing cultural norms. Workers frequently identified gaps between what they were supposed to do and the available resources for achieving it, pointing to problems with equipment, staffing, infrastructure and management as well as poorly designed and poorly functioning micro‐systems (Aveling *et al*. 2015). Though the material deprivations were far more pronounced in the African sites, the nature of the pressures was often strikingly similar, such that the differences were of degree rather than of kind:

Sometimes you don't find anaesthetic drugs, sometimes you don't find stitches, sometimes you don't find oxygen, sometimes you don't find gloves. [Doctor, Sukutra]

They need to intubate a child, but they've only got one laryngoscope and it's not a small one suitable for children. The senior anaesthetist says ‘you see? … that's what happens here, this is ridiculous. Apparently we're supposed to make do'. [Observation, Farnchester]

Pellegrino's stipulation that an organisational and systemic context supportive of safety should exist was therefore frequently violated. One response to these challenges involved individuals in seizing and exercising responsibility. We routinely witnessed staff keeping patients safe in the face of organisational failings by working extremely hard, finding creative solutions or ‘working around’ defects. For example, neonatal units in the African sites frequently lacked sufficient continuous positive airway pressure (CPAP) machines for the number of infants who needed breathing assistance; on their own initiative, local staff had fashioned home‐made devices. In English neonatal units, nurses reported informally re‐organising their work to compensate for the gaps when staff shortages occurred:

You should always do hourly [observations] and generally we're quite good at working as a team so that if you're busy and you don't get to do it then somebody will come and do it for you […] there's been cases in the past where we've been ridiculously busy and somebody has sorted somebody else's patient. [Nurse, Greenborough]

These and other examples are, as Pellegrino suggests, illustrations of how the moral agency of individuals may be required to maintain the border between preventable and non‐preventable harm. But sometimes the norms of practice were not so supportive of safety. The adverse nature of the environments in which people worked and the sheer volume of hazards that had to be negotiated had generalised effects, leaving many staff feeling overwhelmed, depleted and demoralised:

‘Pray for me that I can find another job and leave this place’, said one of the nurses. [Observation, Nikalele]

The nurses talked about how this was really not what they signed up for. They talked about getting other jobs in shops. [Observation, Farnchester]

Staff felt deprived of control and, accordingly, perceived that their agency was limited. On occasion, this meant that they did not appear to accept or to exercise personal responsibility, even when to do so seemed possible and might have secured a better experience or outcomes for the patient. For instance a frail, elderly patient who fell partially out of bed in an English hospital was left hanging in some distress for several minutes while staff nearby completed cleaning another bed, despite being made aware of the situation. In one of the African hospitals, administration of fluids and antibiotics to a very sick baby was delayed by several hours. When a senior doctor asked a junior doctor why this had happened, the junior doctor at first replied that the parents had not brought the drugs, then that the drugs were out‐of‐stock. A few minutes of investigation by the senior doctor found the drugs nearby. The junior had put little effort into finding them, and then given excuses: his sense of personal responsibility was conditioned by a norm that ‘nothing is ever available here’. In the African sites, students and trainees sometimes attempted tasks well beyond the limits of their competence, either in order to gain experience or because no‐one else was available, and they sometimes made serious errors that harmed patients.

These examples appear to stumble on Pellegrino's requirement that each individual health professional must possess the competence and character crucial to the performance of her particular function. But they also show that local norms did not reinforce and sustain these individual competencies and virtue, suggesting that the conduct and behaviour of any individual needs to be understood in its social context. Importantly, these local norms were profoundly (though not exclusively) shaped by the wider socio‐material, economic and historical context. For example, in all hospitals staff‐to‐patient ratios were repeatedly identified as the most important influence on quality and safety, but they were rarely within the control of individual units or even hospital administrators because of externally‐set funding levels and the restricted availability of trained healthcare workers.

In the African hospitals, apparently invincible material deprivations, a sense that access to any care (regardless of its quality) was an improvement, and a background context of high (albeit improving) rates of infant mortality and tragic historical events also influenced normative expectations of standards of care. Thus, death and suffering were sometimes seen as normal and unavoidable, and the space for feasible action–and personal responsibility–accordingly shrunk:

I say [to doctor], ‘I'm sorry to hear you have had a few deaths this week’ and he said ‘yes, but they were all already very poor prognosis babies’. Basically he was saying they weren't avoidable. But some visiting doctors who were working in the unit at the time told me they felt there were in fact avoidable deaths (e.g. one baby died after being in an incubator that wasn't switched on). [Observation, Nikalele]

The deeply ingrained nature of guiding norms meant that some actions and omissions were not readily visible to some staff as violations of standards for which they should have to account. A rather different problem arose when staff felt that they had little choice about their actions, even when they knew the right thing to do. Much concern was reported by participants about the fairness of devolving blame to specific teams and individuals when the problems were felt to originate in external contexts over which they had no control. Several African senior managers, for example, protested that their hospitals were not responsible for failings of care when the reasons for those failings – including a refusal to appoint more staff or to allow patient admissions to be limited – lay with regional or national level authorities. Managers in the English hospitals similarly protested that they were constantly forced to function within externally imposed restrictions, targets, and processes that frustrated rather than assisted them in creating the conditions of safety (McKee *et al*. 2013).

I don't think anybody has the right to penalise us. [Senior manager, Sukutra]

There are many deaths, every day death, and every day it is really hurting. We receive children in numbers beyond our capacity. If the Director does not decide to limit [the number of admissions] when we have exhausted our capacity, what can we do? [Nurse, Nikalele]

I asked can you talk to anybody about that [staffing levels] and she said she could tell the nurse manager but it just goes nowhere. She said the powers that be just don't listen. [Observation, Farnchester]

For workers at all levels, externally‐imposed deprivations of control contributed to a feeling of restricted autonomy and a pervasive sense that it was unreasonable and unfair to hold individuals personally to account. This was a highly consequential problem because, as Pellegrino specifies, affirmation of the moral duty and accountability of each individual in the system is important to patient safety.

All five sites did feature forums which, in principle at least, could call individuals to account, pose questions and pass judgement (Bovens 2007). They were of multiple types, both internal and external to organisations, and were of varying degrees of formality – ranging from peers and colleagues through to external bodies with formal duties of oversight. Some of these external accountability forums, including regulators or accreditation agencies with powers to set standards, inspect, and take action, were of fairly recent origin, particularly in the African sites.

Positive effects of such forums were evident throughout our case studies. Externally‐set standards, targets or goals were sometimes important in helping to remake norms about what was acceptable and to mobilise organisational commitment, largely by setting clear and explicit standards and then holding organisations to account for meeting them. In the English hospitals, these effects were clearly seen, for example, in relation to healthcare‐associated infections:

They started to gradually release Department of Health documents and guidance that was meant to drive organisations to improve standards … the Health and Social Care Act is a must‐do, because it's an Act of Parliament and [organisations] can be legislatively penalised so that really governs the work that we do. [Infection control manager, Farnchester]

The need to account externally not only mobilised changes in organisational procedures, it also helped to signal and reinforce organisational and professional norms by motivating those at the senior levels of hierarchies to take action. For instance, in the African hospitals, a new accreditation requirement to hold and record regular morbidity and mortality (M&M) meetings gave impetus and standing to forums that held individuals to account for standards of professional integrity and competence. At one African M&M meeting, a case concerned twin babies who died because of failures to recognise and act on signs of worsening sepsis caused by the illegitimate absence of a doctor from scheduled duty. In this case, the senior doctor leading the meeting declined to attribute blame to the system, identifying it instead as a lapse in professional duty and thus as one of Bosk's unforgivable errors:

The local senior doctor says ‘so the issue is the doctor who did not round on Sunday. Diseases don't go to pray on Sunday do they? The doctor on duty was not there to do the ward round’. He then adds that ‘you need to do your part. The system may have problems, but you must do your part'. [Observation, Nikalele]

We also found, however, that the available accountability forums – whether internal or external – did not always support the positive affirmations of individuals' duties and accountabilities. The different forums sometimes competed, conflicted, failed to cohere or gave rise to underlaps and overlaps. Whatever form they took, these forums sometimes tolerated poor practice and conduct when they lacked the authority, will, or capacity to act. These failures often occurred at the managerial level:

At the moment there is no follow up … so I can create an action plan but no‐one other than me will take responsibility for coming back and saying well did you achieve what you said you would? [Nurse manager, Worpford]

When forums were absent or failed to impose consequences, some aspects of care tended over time to become normed as optional or to be sloppily performed. For instance, use of a surgical safety checklist was mandated in England by the government in the UK and by the accreditation frameworks used in the African countries. In one African site, the absence of a forum responsible for monitoring its use and the inability of individual nurses to challenge (high status) physicians meant that sanctions for non‐use or poor use were not applied. Ultimately the site abandoned the checklist altogether.

In all settings, forums that might have promoted individual accountability were thwarted because career development and job security depended crucially upon maintaining relationships with senior figures, often creating oppressive conditions where individuals were fearful of the personal consequences of any attempts to ‘speak up’ to superiors or challenge peers about their concerns. Managers in all settings reported that they often felt limited in their power to discipline or control clinical staff – especially senior physicians. In part, this was because of lack of leadership capacity, but it was also linked to the dependence of managers on the cooperation of those they seek to manage (Harvey *et al*. 2014). In the African hospitals, managerial willingness to take unpopular action against highly‐qualified staff was further undermined by the ability of such staff to move elsewhere given skill shortages nationally. Medical students and trainees – who provided most of the day‐to‐day medical care – were accountable to senior, fully qualified doctors, yet those senior doctors were only rarely present in clinical areas. Students and trainees did not always demonstrate the competence or character necessary to secure the safety of their patients, but – in contrast to the sanctioning behaviours of the attending physicians in *Forgive and Remember* and in contradiction of Pellegrino's requirements *–* the system did not reinforce nor sustain these individual competencies and virtues:

Students are doing the cases that should be done by seniors in this hospital. Then afterwards, patients develop infections. When this happens, there is no one that questions the doer. There is no accountability. [Midwife, Sukutra]

In one African site, the institutional arrangements for accountability were especially problematic. Here, the university ran the hospital. Employed by or educationally dependent on the university, physicians and students were accountable to the university, not the hospital's management. One consequence was that training needs were routinely prioritised over patient care and safety. This was evident in the persistent over‐crowding of clinical areas by students, which made it difficult to perform procedures safely, control infection, or respect patients' privacy. Midwives, nurses and others who protested found it difficult to secure cooperation because they could not call students to account, and thus could not function as a forum:

When one mother delivers there are a lot of people who stand there and attend … Students from all batches attend. When you tell them to leave the ward, they refuse. [Midwife, Sukutra]

In principle the exercise of personal moral responsibility might resolve the problem; students could simply vacate crowded spaces or organise themselves to attend in smaller groups. That they did not do so might be seen as ‘weakness of the will’ (Akrasia), but perhaps is better treated as evidence of how behaviours were institutionally organised and structured by the opportunities provided to individuals to be ‘good’: the organisation did not provide alternative ways of being educated, and the behaviour was heavily normed. In Pellegrino's terms, this amounts to a failure to cultivate the conditions of virtuous conduct amounting to constraints on choice of action, but Pellegrino would not rule out a role for personal responsibility altogether. Indeed, sufficient individuals choosing a more virtuous path might be enough to reform the norm, so that a collectivity would act back on the system and in so doing provoke change.

We also found that some accountability forums had negative effects, particularly when the environment was excessively harsh and punitive, when the consequences seemed arbitrary or applied to the wrong parties, and when norms of justice and fairness were violated. Across all hospitals, participants reported didactic, authoritarian, and sometimes aggressive, bullying behaviour by senior colleagues that blamed individuals for problems over which they had little control:

At the end of the case presentation the senior doctor says ‘do you have any questions? What have we discussed?! Eh? Eh?! (aggressive voice). If you've learned nothing then go to the streets!’ [Observation, Nikalele]

Bullying is probably the right word actually, there's a big network of very very senior managers that are just driven about targets. [Nurse manager, Greenborough]

Individuals often lacked confidence that there would be some predictable and fairly applied due process in the event of something going wrong. In African sites, participants reported concerns that incidents would be investigated by agencies lacking the necessary knowledge and skills (e.g. the police). Again consistent with Giddens's argument about the triumph of local norms over external rules and mandates, participants in all sites feared that much of the real power lay outside the formal structure, and that informal consequences frustrated the operation of the formal processes and procedures or (at a minimum) undermined their purpose:

I have talked to someone [who tried to take disciplinary action], there was a sort of harassment directed towards him. At the end of the day you are an individual […] if you have somebody who is dependent on you, it's very difficult to go ahead with such type of confrontation. [Doctor, Sukutra]

You only have to look around where any organisation in the NHS will hound whistleblowers, because they make the organisation look bad […] and if staff put their heads above the parapet and start to try and make a noise and say ‘we're concerned’, they get their heads blown off. [Doctor, Greenborough]

The sense of vulnerability was exacerbated by lack of confidence in the transparency, predictability and fairness of processes associated with patient complaints. In African hospitals, staff particularly feared intimidation by patients' families, unjust or corrupt prosecutions, and lack of access to legal advice or poor support from their employers:

Corruption is the commonest problem everywhere, be it legal system or medical system, it's everywhere. [Doctor, Sukutra]

Because students and trainees in the African hospitals feared being humiliated, suffering educational penalties at the hands of their superiors, or being drawn into potentially corrupt or non‐transparent legal actions, they were sometimes reluctant to acknowledge errors and failures of care both in documentation and when reporting to seniors. As a result, what they reported or documented in patient records was not always what had actually happened. In UK sites, while staff had more confidence in the predictability and resources for medico‐legal procedures, some nonetheless reported that fears of being bullied and of damage to their career prospects inhibited their giving voice to safety concerns.

## Discussion and conclusions

Our analysis provides empirical support for understanding patient safety both as contingent on the dynamic, emergent and recursive duality of structure and agency in healthcare settings and as the outcome of collective effort. It shows, consistent with practice theory, that each individual in a healthcare system typically makes a causal contribution (however small) to outcomes, at the same time as the system shapes and structures the possibilities open to individuals. Across the five sites in our study, the availability of logistical support and resources and the prevailing cultural discourses or norms promoted, enabled or discouraged certain behaviours and practices in particular settings. In consequence, systems and individuals co‐constructed the conditions of safety; each element acted on the other and was mutually constitutive. Our work affirms the ethical principle that individual moral responsibility for actions and behaviours is an irreducible element of professional practice, but also shows empirically that opportunities to ‘be good’ are institutionally organised and structured and that individuals make a crucial contribution towards the creation and reproduction of the normative conditions and criteria to by which they and their actions are to be held account. Without individuals assuming personal moral responsibility and exercising agency, getting the work done in healthcare and getting it done safely both become impossible. We propose the system/individual distinction that has dominated debates about patient safety is in fact a reification: individuals are not somehow ‘outside’ and separate from the system, since they create, modify and are subject to the social forces that are an inescapable feature of any organisational system.

Some safety problems in our case studies were examples of straightforward moral failings, and these actions or omissions by individuals – such as illegitimate absence from scheduled duty – were blameworthy. As both Thompson and Pellegrino make clear, such culpable failures cannot be justified by reference to the context, notwithstanding the evident challenges of those contexts. And, as Bosk argues, in such instances the exercise of social control through sanctions and other means of reinforcing professional norms is essential to protecting patients from potential harm in the long‐run as well as to the maintenance of professional community. At the other end of the spectrum, situations where there was no scope for personal responsibility were also generally clear and unambiguous. Much more common were situations where some space – albeit protean in form – for personal responsibility remained: the interdependence of individual behaviours and organisational and systemic contexts was repeatedly evidenced. Individuals often triumphed in the face of adversity through the exercise of a morally‐founded agency. Where apparently unjustified failures to act on moral responsibility occurred, it seemed that the conscious choices and actions of individuals were heavily conditioned by strongly reinforced norms and other constraints, some of them deeply institutionally and historically patterned.

Participants at all levels of organisational hierarchies were frequently working in settings that not only failed to enable them to provide safe care but also failed to cultivate the conditions of virtuous conduct. Patterned norms and routines acted as signals of what was acceptable practice. As we argued earlier, an important consequence of this is that the perceptions of local actors may be an unreliable guide to questions of moral responsibility or the moral content of actions or omissions: the most insidious form of power ‘consists of letting people whose business it is define what that business includes, which versions of it are serious and important, and which don't matter much.' (Becker 1995: 307). Deeply institutionalised norms and routinised forms of justification may be used both to promote excellent care and to legitimate or obscure poor practices that can harm patients (Dixon‐Woods 2010). Many norms of acceptability and excusability were functional for hard‐working and over‐stretched staff (Dixon‐Woods *et al*. 2009), but they were also sometimes implicated in allowing staff to externalise blame and to attenuate personal responsibility. Some norms rendered some problems – such as harm and assaults on dignity – as normal, natural troubles that were either invisible or inescapable given the circumstances of provision (Dixon‐Woods *et al*. 2011). This in turn had the effect of depressing aspirations and normalising low expectations for quality of care, so that opportunities to improve care even when it was possible to do so were neglected. These findings illustrate the value of a more formal, principles‐based framework for adjudicating on matters of personal responsibility and accountability in order to avoid a descent into unhelpful relativism. But they also underline how each individual contributes to the reproduction of norms about acceptable practice and the important responsibilities associated with that.

Importantly, this principle applies to every individual in an organisation, from the blunt end to the sharp end. Those at management and leadership level have moral agency and moral responsibility, but, importantly, they may be subject to the same supports and inhibitors as those on the shop‐floor. But an emphasis on the personal responsibility of managers and senior figures (internal and external to organisations) is needed to avoid bracketing these individuals as somehow part of ‘the system’ or and to avoid seeing patient safety as the sole responsibility of those at the sharp end. This is especially true given that some people are much more in a position to help cultivate the virtues of others; they may, for example, set the moral tone, model the values, or create and operate accountability processes. Thus, when organisations are not able to increase resources, leaders and managers retain an important role in setting and maintaining expectations of what can be done within the practical limits and providing the context in which the relevant professional virtues can be exercised.

A similar argument might be made in relation to formal standards of practice. If actors are to be made accountable, then the standards they need to meet must be defined and they must be aware of their responsibilities for meeting them. Yet we found workers were not always aware of the relevant standards. As Thompson (2014) notes, ignorance counts as an excuse only if the ignorance is not negligent. Across the five hospitals, lack of awareness was often organisationally induced, and might properly be understood as failures of organisations to provide clarity and enable or support compliance. In many such circumstances, holding individuals to account would not be fair, ethical or effective. But in other instances, it would be reasonable to require individuals to know what they did not know, and that they should not, for example, attempt complex surgery beyond the limits of their competence. This too relies on the clear articulation of standards, expectations and priorities, the consistent evidencing of these in organisational routines and practices, proper attention to them by organisational actors, and oversight by accountability forums.

Our findings suggest that accountability forums (both formal and informal) have the potential, as Pellegrino emphasises and Thompson implies, to improve systems and to influence norms of ‘good’ behaviour. We observed several encouraging examples of such forums, for example when they renewed or created understandings of the proper standards of care and of professional integrity and moral responsibility. Yet these effects were rarely straightforward or easy to achieve, and the forums often failed or had perverse effects. Accountability forums sometimes failed altogether because of weaknesses in leadership or capacity or because informal social systems and quasi‐autonomous professional groups subverted formal systems. Thus, some morally culpable failures escaped with impunity and further reinforced unhelpful norms. Second, individuals feared that due process would not be served, that they would be blamed unfairly and unpredictably (for example for systems problems outside their control) and that the consequences imposed would be harsh and arbitrary. And some accountability forums faltered because many features of standards and answerability were not codified in the mechanisms and processes of accountability systems.

The normative, interpretive work entailed in calling to account was influenced by interrelated material, institutional and symbolic contexts. Thus, when forums sought to impose accountability frameworks underpinned by values of learning and improvement in contexts characterised by authoritarianism or punitive responses to errors, the clash of values created confusion of purpose. This, together with overwhelming structural constraints, weak systems for control and oversight, perceptions of corruption and nepotism in some contexts, and absence of follow‐through when problems were detected, fuelled apathy and fatalism. The effect was to continually erode the morale, energy and will of staff and the distinction between genuinely insurmountable problems and less legitimate excuses.

Our analysis demonstrates the need to be sensitive to wider institutional contexts beyond both specific organisations and beyond healthcare. Many constraints on organisations and individuals arose institutionally. Individual organisations were rarely fully in control of their own destiny: budgets, resources, targets and policies were often externally imposed by regional or national government agencies. More broadly, in the African hospitals, the wider context undermined workers' beliefs that the legal systems and institutions of state would operate fairly and impartially, and that it was possible to survive personally by always acting in the collective interest.

## Conclusions

These findings furnish empirical evidence of a basic sociological tenet of practice theory – the interdependence of systemic and individual agency at all levels – in the context of patient safety in healthcare systems. An uncritically‐applied ‘no blame’ approach may fail to recognise variations in the type and scope of opportunities for individuals to assert their moral responsibility, but a calculus‐like logic seeking to promote ‘just culture’ that fails to recognise the limits of individual autonomy and the messiness of standards of practice may be equally misguided. Individual agency is both an ethical requirement and a means of modifying systems themselves; never holding individuals to account risks normalisation of failure, fatalism, externalisation of blame and apathy, and may erode collective commitments and values. The ability to impose sanctions for culpable failures is likely to remain an important feature of any well‐functioning accountability system, but legitimate and effective exercise of this ability depends on a predictable, fair and effectively operated institutional infrastructure, with proportionate consequences and alignment of the values and processes of different internal and external forums.

It is not a matter of balancing systems against individual accountability; instead, their recursive nature needs to be recognised. The opportunities for workers to ‘be good’ are made logistically possible and cultivated culturally both by the organisations they work in and by wider institutional structures. Accordingly, the interdependence of institutional and socio‐material and economic contexts (within and beyond the hospital) needs to be taken into account in the design of accountability frameworks. Systems need to be designed and operated to support, cultivate and sustain individual competence and virtue, with explicit attention to how they encourage the norms and values that shape the exercise of moral agency. We conclude that an important responsibility both of organisations and accountability systems is the cultivation and enabling of individuals' moral agency and the fostering of the conditions of moral community. Making accountability ‘work’ for patient safety is not simply a question of designing the perfect algorithm for blame distribution; one of the major obligations of healthcare organisations (and wider institutions) in relation to patient safety is to nurture the conditions of moral community. Achieving this will require deep understanding of processes of social control.
